# The tumour sink effect on ^68^Ga-PSMA-PET/CT in metastatic castration-resistant prostate cancer and its implications for PSMA-RPT: a sub-analysis of the 3TMPO study

**DOI:** 10.1186/s40644-025-00910-z

**Published:** 2025-07-15

**Authors:** Atefeh Zamanian, Étienne Rousseau, François-Alexandre Buteau, Frédéric Arsenault, Alexis Beaulieu, Geneviève April, Daniel Juneau, Nicolas Plouznikoff, Éric E. Turcotte, Catherine Allard, Patrick O. Richard, Fred Saad, Brigitte Guérin, Frédéric Pouliot, Jean-Mathieu Beauregard

**Affiliations:** 1 Department of Radiology and Nuclear Medicine; and Cancer Research Centre, Université Laval, Quebec City, QC Canada; 2 Oncology Axis, CHU de Québec - Université Laval Research Centre, Quebec City, QC Canada; 3 Department of Nuclear Medicine and Radiobiology, Université de Sherbrooke, Sherbrooke, QC Canada; 4 Sherbrooke Molecular Imaging Centre, Centre de recherche du Centre hospitalier universitaire de Sherbrooke, Sherbrooke, QC Canada; 5 Division of Nuclear Medicine, Department of Medical Imaging, CHU de Québec – Université Laval, 11 Côte du Palais, Quebec City, QC G1R 2J6 Canada; 6 Department of Radiology and Nuclear Medicine, Centre Hospitalier de l’Université de Montréal, Montreal, QC Canada; 7 Unité de recherche clinique et épidémiologique, Centre de recherche du Centre hospitalier universitaire de Sherbrooke, Sherbrooke, QC Canada; 8 Division of Urology, Department of Surgery, Centre Hospitalier Universitaire de Sherbrooke, Sherbrooke, QC Canada; 9 Division of Urology, Department of Surgery, Centre Hospitalier de l’Université de Montréal, Montreal, QC Canada; 10 Division of Urology, Department of Surgery, Université Laval, Quebec City, QC Canada

**Keywords:** PET/CT, Prostate cancer, Prostate-specific membrane antigen (PSMA), Radiopharmaceutical therapy, Sink effect, PSMA-617, Total lesion fraction (TLF)

## Abstract

**Background:**

The tumour sink effect is a phenomenon whereby the sequestration of a radiopharmaceutical in cancer lesions leads to decreased activity concentration in the blood stream and organs. The aim of this sub-analysis of the prospective 3TMPO study (NCT04000776) was to investigate the tumour sink effect on prostate-specific membrane antigen (PSMA) PET imaging in a population of patients with metastatic castration-resistant prostate cancer (mCRPC).

**Methods:**

Ninety-seven participants underwent ^68^Ga-PSMA-617 PET/CT imaging. The activity concentration in the kidney, parotid, spleen, liver and blood was expressed as a percentage of injected activity per cubic centimetre (%IA/cm^3^). The total tumour volume was delineated, and the total lesion fraction (TLF), i.e., the percentage of injected activity sequestered in the tumour, was computed. Participants were stratified into three tumour burden groups: small (TLF < 10%), moderate (10% ≤ TLF < 25%), and large (TLF ≥ 25%). Weight, lean body weight, body surface area, and estimated glomerular filtration rate (eGFR) were investigated as additional factors affecting biodistribution.

**Results:**

The TLF ranged from 0.0 to 43.5%. For all healthy tissues, the %IA/cm^3^ was negatively correlated with TLF (*r* ranging − 0.33 to − 0.46; *P* < 0.001). Patients with a large TLF had significantly lower uptake in all organs when compared to those with a small TLF (*P* < 0.05). Body habitus indices and/or eGFR were negatively correlated with the %IA/cm^3^ of the parotid, liver and blood (*r* ranging − 0.23 to − 0.33; *P* < 0.05). Combining predictive variables, the term [BSA / (1–TLF)] tended to yield the strongest negative correlations with healthy tissues %IA/cm^3^ (*r* ranging − 0.33 to − 0.63; *P* < 0.001).

**Conclusion:**

The tumour sink effect was observed in a cohort of mCRPC patients scanned with ^68^Ga-PSMA-617. This finding strongly suggests that patients with a large TLF are likely to receive lower absorbed doses to organs at risk – i.e., be undertreated from a dosimetry perspective – following a fixed-activity regime of ^177^Lu-PSMA-617 radiopharmaceutical therapy, as commonly practiced. Individual factors such as body habitus and renal function further impact the biodistribution of PSMA radiopharmaceuticals.

**Trial registration:**

NCT04000776, registered on 2019-06-27.

**Supplementary Information:**

The online version contains supplementary material available at 10.1186/s40644-025-00910-z.

## Background

Radiopharmaceutical therapy (RPT) of metastatic castration-resistant prostate cancer (mCRPC) based on targeting prostate-specific membrane antigen (PSMA) utilizing ^177^Lu-PSMA-617 has been shown to prolong progression-free, quality-of-life deterioration-free, as well as overall survival, and has gained regulatory approval in many jurisdictions [1, 2]. However, the complete remission rate is dismal, and many patients do not experience significant anti-tumour effects, e.g., prostate-specific antigen continuously rises under treatment and/or pain is not relieved. In our experience, this tends to happen more often in cases of extensive metastatic disease, and others have shown that patients with large tumour volume have poor survival despite receiving PSMA RPT [3]. In these patients, the sequestration of a radiopharmaceutical by the tumour may contribute to reduce the uptake and thus the absorbed doses to healthy tissues, a phenomenon commonly referred to as the *tumour sink effect* [4]. Furthermore, it has been shown that for ^177^Lu-based PSMA RPT, the specific absorbed dose (i.e., Gy/GBq) to organs at risk varies considerably between patients, often by one order of magnitude or more [5, 6]. The currently approved fixed-activity ^177^Lu-PSMA-617 regime (i.e., six cycles of 7.4 GBq) being well tolerated implies that most patients receive absorbed doses below the tolerance threshold, and those affected by the tumour sink effect would tend to be further undertreated with such a one-size-fits-all regime.

The 3TMPO study was a multicentre, prospective study in patients with mCRPC investigating intermetastasis heterogeneity using ^68^Ga-PSMA-617, ^18^F-fluorodeoxyglucose (FDG) PET/CT and, in select participants, ^68^Ga-DOTATATE PET/CT (NCT04000776) [7]. Thus, this cohort constituted a unique opportunity to study the tumour sink effect on PSMA PET/CT. While other groups have reported on this topic, their analyses were limited to traditional PET metrics, such as molecular tumour volume (MTV), which only partially reflect tumour sequestration; as well as total lesion activity (TLA) and standardized uptake value (SUV), both of which are biased by body weight [8–13]. In an approach faithful to the sink effect concept as we originally introduced it for modern theranostics, we conducted this sub-analysis of the 3TMPO study using metrics that are more directly relevant to RPT—the total lesion fraction (TLF) and percent injected activity per cm^3^ (%IA/cm^3^)—and taking into account the influence of other patient factors known to affect PSMA radiopharmaceutical biodistribution [4, 5, 14, 15].

## Methods

### Study population and imaging

The 3TMPO study was approved by a central ethics committee and all participants signed a written consent form. Key eligibility criteria included a history of progressive mCRPC and the presence of at least three metastases on conventional imaging (CT and bone scan) [7]. This sub-analysis is limited to the ^68^Ga-PSMA-617 PET/CT. In brief, the latter was performed from at least mid-skull to proximal thighs 60 min after the injection of 1.8–2.2 MBq/kg of ^68^Ga-PSMA-617, with a low-dose CT scan. Using NEMA phantom acquisitions, the multiple PET/CT scanners of various makes and models that were used across the five participating centres were assessed for quantitative accuracy, and reconstruction parameters were standardized to reduce variability in image quality.

### Healthy tissue activity sampling and tumour segmentation

Using Encore software (MIM Software Inc., Cleveland, Ohio, USA), 3-cm circular volumes of interest (VOIs) were placed in the centre of the right liver lobe, avoiding any metastases, and in the ascending aorta. For the spleen, kidney, and parotid gland, 2-cm VOIs were centered on the location of the SUV_peak_ of each organ (highest SUV_peak_ across both sides for the kidneys and parotids). Activity concentration was primarily expressed as %IA/cm^3^, and secondarily as SUV_mean_. Whole-body MTV was segmented using an SUV threshold of 1.5x the liver SUV_mean_. Then, sub-VOIs, each < 1 cm^3^ in volume, as well as any area of uptake classified as physiological or likely benign, were removed. In addition to MTV and tumour SUV (max, peak, and mean), TLA and TLF (TLA divided by body weight in grams, expressed as a percentage) were computed, the latter constituting our primary tumour burden metric [15]. All reported SUV and TLA data were normalized for body weight. Participants were stratified into three tumour burden groups: small (TLF < 10%), moderate (10% ≤ TLF < 25%), and large (TLF ≥ 25%). We also conducted the same analysis as Gafita et al. [11], stratifying participants by MTV quintile.

### Body habitus and renal function

The body habitus variables (i.e., surrogate volumes of distribution affecting uptake in healthy organs) of weight, lean body weight (LBW; James formula), and body surface area (BSA; Mosteller formula) were considered [16, 17]. Estimated glomerular filtration rate (eGFR; mL/min./1.73 m^2^) was determined from the serum creatinine level (µmol/L), using the CKD-EPI and MDRD-4 formulas [18, 19]. The creatinine clearance rate was computed using the Cockroft-Gault equation [20].

### Statistical analyses

Continuous variables were reported as median, interquartile ranges (IQRs) or mean and standard deviation (SD) range. The relationship between organ uptake and tumour burden was assessed using Spearman’s correlations. We also examined the differences in organ uptake among categorized tumour burden groups using the Kruskal-Wallis test followed by Dunn’s post-hoc test with Bonferroni correction. Statistical analyses were performed using Prism (v. 9.4.1; GraphPad Software, La Jolla, CA, USA). *P* values of 0.05 or less were considered statistically significant.

## Results

### Participant population

Ninety-eight eligible mCRPC participants underwent a ^68^Ga-PSMA-617 PET/CT as part of the 3TMPO study. One participant was excluded because of missing data (creatinine to calculate eGFR); thus, 97 participants were included in this analysis (Table 1).

**Table 1:** Participant characteristics (*n* = 97)

	Mean $\:\pm\:$ SD (range) or Median [IQR] (range)	*n* (%)
Age (y)	69.3 ± 7.4 (44–87)	
Height (cm)	170.9 ± 6.8 (156–193)	
Weight (kg)	83.4 ± 12.7 (59–130)	
LBW (kg)	60.7 ± 5.9 (47.3–79.8)	
BSA (m^2^)	1.98 ± 0.17 (1.61–2.58)	
eGFR (mL/min/1.73 m^2^)	86.7 ± 11.7 (54.6–120.2)	
PSA (µg/L)	51.1 [20.8–206.0] (0.0–2,394.0)	
ECOG PS		
0		38 (39.2)
1		49 (50.5)
2		9 (9.3)
Unknown		1 (1.0)
Number of treatment lines for mCRPC		
0		8 (8.2)
1		20 (20.6)
2		11 (11.3)
> 2		58 (59.8)
Location of PSMA-positive lesions		
Prostate or prostate fossa		15 (15.5)
Lymph nodes		63 (64.9)
Bone		82 (84.5)
Viscera and others		36 (37.1)

BSA = body surface area; ECOG PS = Eastern Cooperative Oncology Group performance status; eGFR = estimated glomerular filtration rate; IQR = interquartile range; LBW = lean body weight; mCRPC = metastatic castration-resistant prostate cancer; PSA = prostate-specific antigen; PSMA = prostate-specific membrane antigen

### Tumour burden and healthy tissue uptake

There was a very high interpatient variability in tumour uptake (SUV and %IA/cm^3^; Table 2, Supplemental Fig. 2) and tumour burden (MTV, TLA, and TLF; Table 2, Supplemental Fig. 1). When stratified according to their TLF, there were 72 participants with a low tumour burden, 17 with a moderate tumour burden, and eight with a large tumour burden. Notably, up to 43.5% of the PSMA radioligand was sequestered in the tumour; thus, it was not available for healthy tissue uptake. The latter also varied considerably among participants, with %IA/cm^3^ and SUV_mean_ ranging by a factor of four to more than tenfold between individuals (Table 3, Supplemental Fig. 2).

**Table 2:** Tumour uptake and burden (*n* = 97)

	Median	IQR	Range
SUV_max_	29.5	18.9–55.5	0.0–218.5
SUV_peak_	19.8	13.1–34.5	0.0–124.9
SUV_mean_	8.7	6.8–11.5	0.0–30.0
Mean %IA/cm^3^	0.011	0.008–0.013	0.000–0.029
MTV (cm^3^)	199	63–801	0–3,750
TLA (cm^3^)	1,793	618–10,197	0–37,919
TLF (%)	2.2	0.8–11.6	0.0–43.5

%IA/cm^3^ = percentage of injected activity per cubic centimetre; IQR = interquartile range; MTV = molecular tumour volume; SUV = standardized uptake value; TLA = total lesion activity; TLF = total lesion fraction

**Table 3:** Healthy tissue uptake (*n* = 97)

		Mean %IA/cm^3^				SUV_mean_	
	Median	IQR	Range		Median	IQR	Range
Kidney	0.024	0.019–0.030	0.010–0.043		20.0	15.7–26.2	6.4–35.1
Parotid	0.010	0.008–0.015	0.003–0.022		8.9	7.0–11.5	2.9–19.7
Spleen	0.007	0.005–0.009	0.002–0.027		5.6	4.5–7.7	1.5–18.9
Liver	0.004	0.003–0.005	0.002–0.010		3.2	2.7–3.9	1.6–6.7
Blood	0.003	0.003–0.003	0.001–0.005		2.6	2.2–2.9	0.9–4.1

%IA/cm^3^ = percentage of injected activity per cubic centimetre; IQR = interquartile range; SUV_mean_ = mean standardized uptake value

### Tumour sink effect

**Fig. 1:**
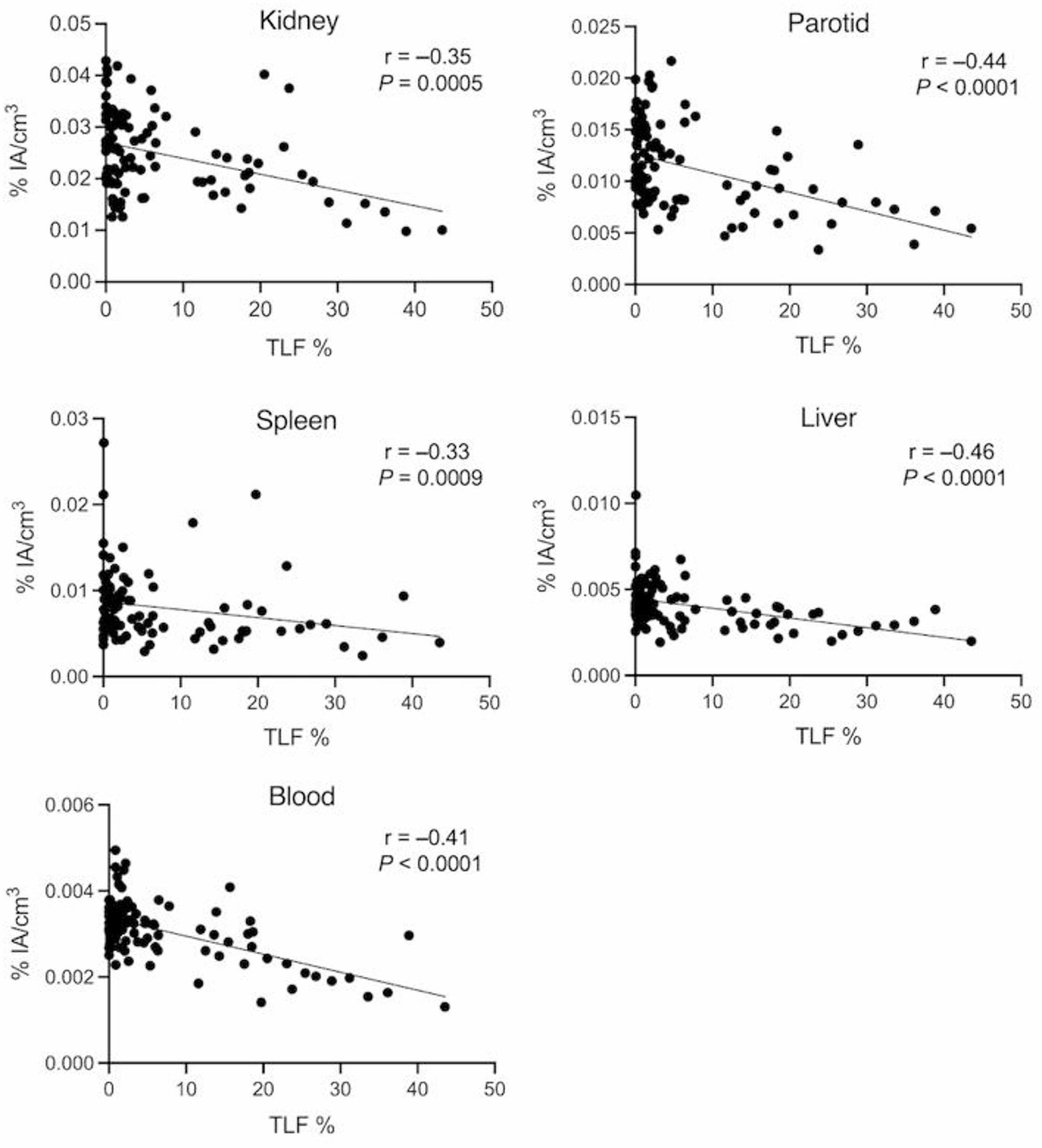
Spearman’s correlations between healthy tissue uptake (mean percent injected activity per cm^3^; %IA/cm^3^) and total lesion fraction (TLF). Linear regression lines are shown for illustrative purposes (*n* = 97)

**Fig. 2:**
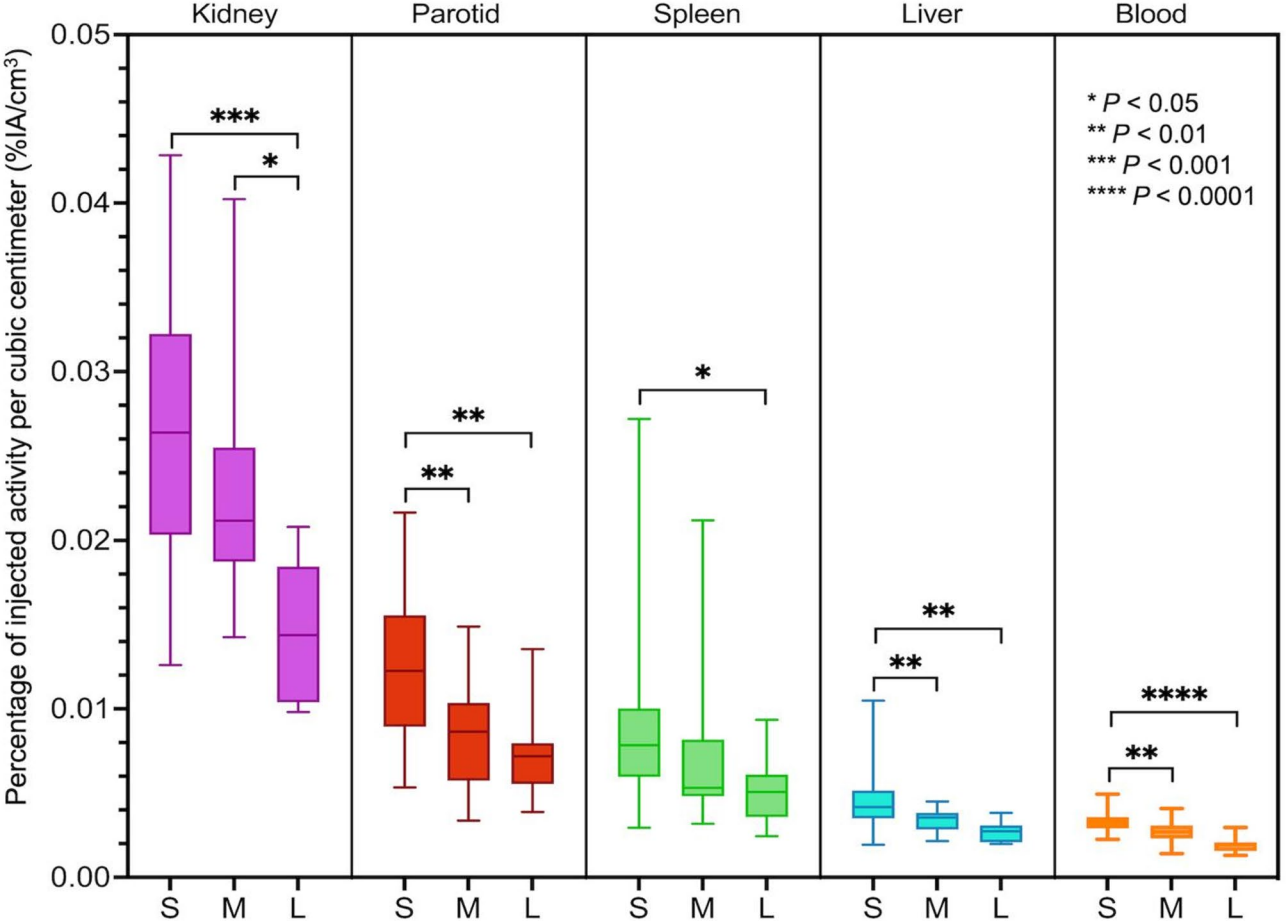
Healthy tissue uptake in participants with small (S: < 10%), moderate (M: 10–25%), and large (L: ≥ 25%) total lesion fraction (TLF). Median, interquartile range (box), and range (whiskers) are shown

**Fig. 3:**
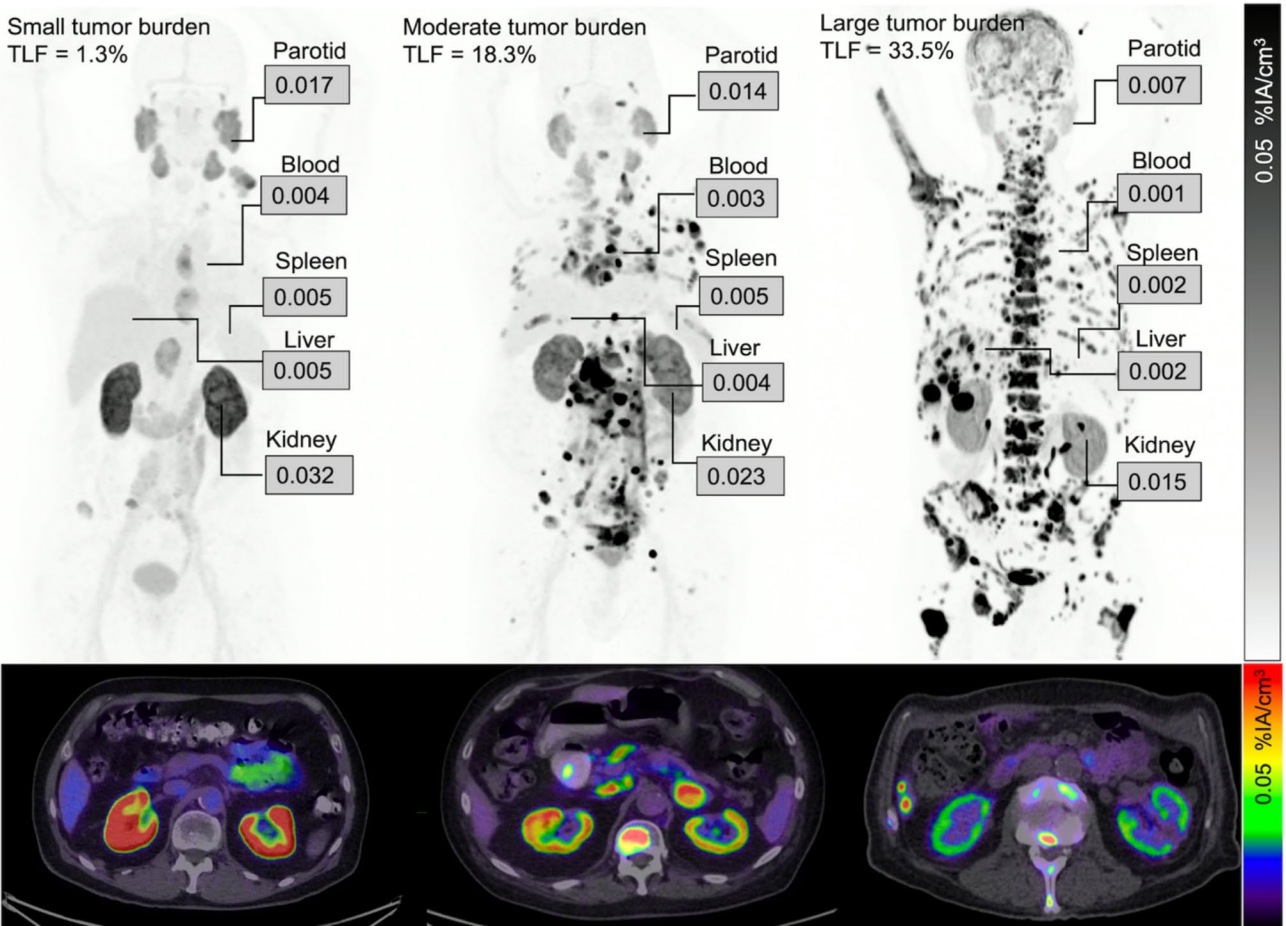
Maximum intensity projections (top row) and fused axial slices (bottom row) of three patients with different total lesion fractions (TLF). Uptake values are shown as mean percentage of injected activity per cubic centimetre (%IA/cm^3^)

There were significant negative correlations between heathy tissue uptake (%IA/cm^3^) and TLF (Fig. 1; Table 4). When grouping participants according to their TLF, there was a statistically lower uptake in participants with a large tumour burden than in those with low tumour burden (Fig. 2). Representative cases are illustrated (Fig. 3).

**Table 4:** Spearman’s correlations between participant characteristics (TLF, body habitus, eGFR, and combinations thereof) and healthy tissue mean uptake (%IA/cm^3^)

	Kidney		Parotid		Spleen		Liver		Blood	
	*r*	*P*	*r*	*P*	*r*	*P*	*r*	*P*	*r*	*P*
TLF	**–0.35**	< 0.001	**–0.44**	< 0.001	**–0.33**	< 0.001	**–0.46**	< 0.001	**–0.41**	< 0.001
Weight	–0.09	0.36	**–0.27**	0.009	–0.16	0.11	**–0.33**	< 0.001	**–0.26**	0.009
BSA	–0.05	0.57	**–0.23**	0.02	–0.17	0.08	**–0.33**	< 0.001	**–0.27**	0.004
LBW	–0.01	0.88	–0.20	0.06	–0.15	0.14	**–0.33**	< 0.001	**–0.28**	0.006
eGFR*	0.07	0.47	–0.17	0.09	–0.11	0.29	**–0.23**	0.02	**–0.30**	0.002
Weight × eGFR	–0.01	0.94	**–0.27**	0.008	–0.16	0.13	**–0.34**	< 0.001	**–0.34**	< 0.001
BSA × eGFR	0.02	0.84	**–0.24**	0.02	–0.17	0.15	**–0.33**	< 0.001	**–0.38**	< 0.001
LBW × eGFR	0.04	0.69	**–0.23**	0.02	–0.16	0.12	**–0.33**	< 0.001	**–0.37**	< 0.001
Weight / (1 – TLF)	**–0.28**	0.006	**–0.50**	< 0.001	**–0.32**	0.001	**–0.57**	< 0.001	**–0.52**	< 0.001
BSA / (1 – TLF)	**–0.33**	< 0.001	**–0.53**	< 0.001	**–0.40**	< 0.001	**–0.63**	< 0.001	**–0.61**	< 0.001
LBW / (1 – TLF)	**–0.31**	0.002	**–0.52**	< 0.001	**–0.38**	< 0.001	**–0.62**	< 0.001	**–0.62**	< 0.001
Weight × eGFR / (1 – TLF)	–0.15	0.13	**–0.43**	< 0.001	**–0.29**	0.004	**–0.52**	< 0.001	**–0.56**	< 0.001
BSA × eGFR / (1 – TLF)	–0.16	0.11	**–0.44**	< 0.001	**–0.30**	0.003	**–0.54**	< 0.001	**–0.60**	< 0.001
LBW × eGFR / (1 – TLF)	–0.15	0.13	**–0.43**	< 0.001	**–0.30**	0.003	**–0.54**	< 0.001	**–0.60**	< 0.001

*CKD-EPI formula

*r* values in bold are statistically significant

BSA = body surface area; eGFR = estimated glomerular filtration rate; LBW = lean body weight; %IA/cm^3^ = percentage of injected activity per cubic centimetre; TLF = total lesion fraction

For the purpose of comparison with other studies, we assessed the correlations between healthy tissue SUV_mean_ and MTV, which also proved to be significantly negative for all tissues: kidney (*r* = − 0.34; *P* < 0.001), parotid (*r* = − 0.45; *P* < 0.001), spleen (*r* = − 0.33; *P* = 0.001), liver (*r* = − 0.58; *P* < 0.001) and blood (*r* = − 0.41; *P* < 0.001). To replicate the analysis conducted by Gafita et al. [11], we stratified participants as controls (when MTV = 0; *n* = 7) and per MTV quintile (*n* = 90) (Supplemental Fig. 3). Trends towards lower tissue SUV_mean_ in participants from the highest MTV quintile were observed, although not reaching statistical significance for the spleen.

### Impact of body habitus and renal function on biodistribution

Uptake by healthy tissues (%IA/cm^3^) was inversely related to weight, LBW, and BSA, with significant to near-significant correlations for parotid, liver, and blood (Table 4). Apart from the kidney, correlation coefficients were negative between other tissues’ uptake and renal function expressed as eGFR (CKD-EPI formula), and statistical significance was reached for liver and blood. Conversely, the kidney uptake at this imaging time point (60 min.) did not seem dependent on the renal function. We explored other indices of renal function as explanatory variables (Cockcroft-Gault creatinine clearance and eGFR using the MDRD-4 formula) but none yielded meaningfully different results (data not shown). When combining indices of body habitus and/or renal function with TLF, we found the strongest relationships with BSA/1–TLF, i.e., the BSA divided by the non-tumoral fraction. Notably, when expressing tissue uptake using SUV_mean_ corrected for body weight, as others have done, the correlations with all body habitus indices turned positive, suggesting that body weight is a biased volume of distribution for SUV calculation with ^68^Ga-PSMA-617 (Supplemental Table 1). Regardless, SUV_mean_ of all tissues was still significantly negatively correlated with TLF.

## Discussion

In a prospective observational cohort of patients with mCRPC at various stages of the disease who underwent ^68^Ga-PSMA-617 PET/CT, we observed a significant tumour sink effect in healthy tissues, i.e., the more the tracer is sequestered by the tumour, the less it is available to accumulate in these tissues. As introduced earlier, we believe that the most significant clinical implication of the tumour sink effect is that, in PSMA RPT, patients with a large tumour burden will likely be undertreated, from a dosimetry perspective, when a fixed activity is administered, such as per the currently approved regime of ^177^Lu-PSMA-617 [2]. Indeed, the tumour sequestration spares organs at risk (i.e., the parotid, the kidney, and the bone marrow, for which blood is often considered a dosimetric surrogate) from radiation exposure by removing a portion of the radiopharmaceutical from the blood stream. Consequently, when they are treated with a one-size-fits-all RPT regime, patients with a larger tumour burden, who tend to be more symptomatic and in greater need of therapeutic efficacy, may not receive the maximum injected activity – hence tumour absorbed dose – that they could tolerate. However, these are the patients who could potentially benefit the most from dosimetry-based, personalised RPT, especially since they have a poor prognosis [3, 21]. Further research validating the tumour sink effect on healthy tissues dosimetry in patients treated with PSMA RPT is warranted, paving the way to clinical trials of personalised PSMA PRT.

The sink effect is not limited to PSMA theranostics. We previously reported on the tumour sink effect in patients with neuroendocrine tumour undergoing ^68^Ga-DOTATATE PET/CT [4]. Our initial results, using a renal dosimetry-based regime of peptide receptor radionuclide therapy with ^177^Lu-DOTATATE, showed that it is possible to safely escalate administered activity and tumour absorbed dose in most patients, including those with larger tumour burden [22]. This approach could be translated to PSMA RPT. However, caution is advised in cases of sink effect resulting from extensive bone metastasis, which is more prevalent in prostate cancer than in neuroendocrine tumour patients. Indeed, while in such cases the bone marrow would receive lower self-dose and cross-dose from the blood, the cross-dose from bone tumour infiltration may be high, counteracting the bone marrow sparing by the sink effect. On the other hand, patients with extensive soft-tissue metastasis (with or without limited bone disease) clearly have the potential to draw more benefits from personalised RPT without risking undue toxicity. Further research is warranted on how to best use PSMA PET (e.g., TLF and/or healthy tissue uptake) to predict specific absorbed doses and, ultimately, to personalise the first cycle of PSMA RPT [23].

Others have reported on the sink effect on PSMA PET and we have summarized key features and results of their studies in Table 5. Unlike other researchers who analysed retrospective series, we derived our results from a prospective cohort. We used ^68^Ga-PSMA-617 in the 3TMPO study, a PSMA radioligand that is less commonly used in the clinics than ^68^Ga-PSMA-11 and ^18^F-DCFPyL, but which has a similar biodistribution [24, 25]. Thus, our results supplement those of others, confirming that the sink effect can be observed with all these urea-based radioligands, including ^68^Ga-PSMA-617, which shares the same pharmaceutical moiety as ^177^Lu-PSMA-617. Others have mainly used the MTV metric, or a visual categorical equivalent, to quantify or stratify tumour sequestration. While MTV is likely the most significant driver of the sink effect, intensity of uptake is the other defining dimension thereof. In this respect, Burgard et al. used TLA, the product of MTV and SUV_mean,_ which is a more comprehensive metric for the purpose [13]. However, both MTV and TLA are contingent on knowledge of body weight to determine the relative magnitude of the tumour burden (e.g., for a given MTV or TLA, the tumour is twice as burdensome for a 50 kg patient than for a 100 kg patient). Instead, we propose TLF as a simplified, more interpretable, and direct metric of the tumour sink effect that is independent of weight and may facilitate interpatient comparisons [15]. In principle, TLF directly represents the proportion by which the healthy tissue uptake would be decreased because of cancer load. As for the metric of organ uptake, we primarily used %IA/cm^3^ rather than SUV_mean_, as others did, because the former is more directly related to the specific absorbed dose (i.e., Gy/GBq, or Gy per a fixed activity) when extrapolating to RPT. Indeed, assuming equal biokinetics, the specific absorbed doses in the tissues of patients of varied body weight will be proportional to %IA/cm^3^ but not to SUV. Notably, all but one group observed a statistically significant relationship describing a tumour sink effect in at least one healthy tissue of interest with direct relevance to PSMA RPT. Werner and al. were the exception and this is likely because their analysis was restricted to patients with low-volume disease (< 100 cm^3^) [12]. Observation of a meaningful sink effect obviously requires inclusion of patients with a large tumour burden.

**Table 5:** Summary of reports of the tumour sink effect on PSMA-PET in prostate cancer for selected healthy tissues

1st author	PET tracer	Methods	Tumour burden variable or groups	Organ uptake variable	Kidney	Parotid	Spleen	Liver	Blood
Gaertner [8]	^68^Ga-PSMA-11	• Retrospective • 135 PC patients • Tumour burden visually rated as low, medium, or high	Medium *vs.* Low	Δ SUV_mean_	–18.4%*	**–31.4%**	–6.9%	–3.4%	0.40%
			High *vs.* Low		**–44.6%**	**–43.4%**	**–18.6**%	**–24.6%**	**–8.1%**
Groener [9]	^68^Ga-PSMA-11	• Retrospective • 85 mCRPC patients • Tumour burden visually rated according to PROMISE • 54 control patients (localized PC)	Oligofocal *vs.* Control	Δ SUV_mean_	N/A	–5.7%	N/A	–5.9%	N/A
			Multifocal *vs.* Control			–**27.1%**		–6.7%	
			Disseminated *vs.* Control			–**20.0%**		–15.1%	
			Diffuse *vs.* Control			–**55.8%**		–**30.4%**	
Tuncel [10]	^68^Ga-PSMA-11	• Retrospective • 65 mCRPC patients • Tumour burden visually categorized as tumour sink effect positive or negative on post ^177^Lu-PSMA-617 planar scan	Positive vs. Negative	Δ SUV_mean_	**–24.9%**	**–27.8%**	N/A	**–29.4%**	N/A
Gafita [11]	^68^Ga-PSMA-11	• Retrospective, multicentre • 356 PC patients • Tumour burden stratified by MTV quintile • 50 control patients (PET negative)	Quintile 1 *vs.* Control	Δ SUV_mean_	–12.6%	–6.3%	–12.7%	–13.6%	N/A
			Quintile 2 *vs.* Control		**–23.0%**	0.50%	–14.9%	**–11.4%**	N/A
			Quintile 3 *vs.* Control		**–23.6%**	–1.8%	–6.3%	**–11.4%**	N/A
			Quintile 4 *vs.* Control		**–30.0%**	**–24.5%**	**–23.8%**	**–15.9%**	N/A
			Quintile 5 *vs.* Control		**–40.0%**	**–38.1%**	**–34.9%**	**–43.2%**	N/A
			MTV	SUV_mean_	***r*** **= − 0.34**	***r*** **= − 0.44**	***r*** **= − 0.16**	***r*** **= − 0.30**	N/A
Werner [12]	^18^F-DCFPyL	• Retrospective • 50 PC patients	MTV	SUV_mean_	*r* = − 0.18	*r* = 0.16	*r* = 0.005	*r* = − 0.17	N/A
Burgard [13]	^68^Ga-PSMA-11	• Retrospective • 33 mCRPC patients • 25 RPT responders • PET post vs. pre RPT	TLA	SUV_mean_	*r* = − 0.17	***r*** **= − 0.44**	***r*** **= − 0.42**	*r* = − 0.34	N/A
			Δ TLA	Δ SUV_mean_	*r* = 0.08	***r*** **= − 0.40**	***r*** **= − 0.36**	*r* = − 0.30	N/A
Zamanian [this study]	^68^Ga-PSMA-617	• Prospective, multicentre • 97 mCRPC patients	MTV	SUV_mean_	***r*** **= − 0.34**	***r*** **= − 0.50**	***r*** **= − 0.33**	***r*** **= − 0.58**	***r*** **= − 0.41**
			TLA	SUV_mean_	***r*** **= − 0.31**	***r*** **= − 0.44**	***r*** **= − 0.33**	***r*** **= − 0.52**	***r*** **= − 0.40**
			TLF	%IA/cm^3^	***r*** **= − 0.35**	***r*** **= − 0.44**	***r*** **= − 0.33**	***r*** **= − 0.46**	***r*** **= − 0.41**

* Data in bold font indicate statistically significant values (*P* < 0.05)

%IA/cm^3^ = mean injected activity per cubic centimetre; mCRPC = metastatic castration-resistant prostate cancer; MTV = molecular tumour volume; N/A = not assessed; PROMISE = *Prostate Cancer Molecular Imaging Standardized Evaluation;* PC = prostate cancer; PSMA = prostate-specific membrane antigen; RPT = radiopharmaceutical therapy; SUV_mean_ = mean standardized uptake value; TLA = total lesion activity; TLF = total lesion fraction

A larger volume of distribution and a faster excretion from the body of virtually any radiopharmaceutical will inevitably contribute to lowering its concentration in blood and tissues. Accordingly, in patients with neuroendocrine tumours, we have found that body habitus and renal function were factors affecting the biodistribution and dosimetry of DOTATATE compounds in tissues of interest including the kidney [4, 26, 27]. In prostate cancer patients, using quantitative SPECT at ~ 48 h after ^177^Lu-PSMA-I&T RPT, we found that body habitus and renal function were both significantly and negatively correlated with specific absorbed dose estimates for the bone marrow and the kidney [14]. In accordance with our data, Violet et al. showed a significant absorbed dose reduction in parotid glands with increasing BSA [5]. In this study, we observed a strong correlation between body habitus indices or eGFR and activity concentration of ^68^Ga-PSMA-617 in the blood (as a surrogate for bone marrow), but not in the kidney. We hypothesize that at an early imaging timepoint (~ 60 min. for PET/CT), more complex and competing rapid kinetics at the renal cell level, superimposed over excreted activity into nephron tubules, may blur the relationships between apparent renal activity concentration and these patient characteristics. Moreover, the correlation between body habitus indices and renal SUV_mean_ was revealed as significantly positive (Supplemental Table 1), pointing at body weight as an overestimated volume of distribution for ^68^Ga-PSMA-617, which does not exhibit a particular tropism for fatty tissues. This also raises concerns about the use of tumour SUV cutoffs for patient selection for RPT (e.g., SUV_max_ ≥ 20 in at least one lesion and ≥ 10 in all other lesions in the TheraP trial) [28], as overweight patients would technically be favoured over underweight patients in terms of access to the RPT. Normalizing SUV by LBW or BSA may help to reduce this bias [29, 30], although the use of a SUV ratio (e.g., uptake relative to the liver) may be preferable as it is independent of body habitus. We are currently conducting more in-depth analyses regarding these issues, which will be the topic of upcoming publications.

## Conclusion

Patients with mCRPC have a wide range of PSMA-expressing tumour burdens, causing a tumour sink effect on ^68^Ga-PSMA-617 PET/CT, which manifests as a decreasing uptake in healthy tissues as the TLF increases. Other factors such as body habitus and renal function further affect biodistribution. This actionable knowledge is highly relevant for PSMA RPT, to which most of these patients are candidate. With the currently approved fixed-activity regime of ^177^Lu-PSMA-617, patients with a large tumour burden are likely undertreated from a dosimetry perspective, while being the most in need of therapeutic relief.

## Electronic supplementary material

Below is the link to the electronic supplementary material.

Supplementary Material 1

## Data Availability

No datasets were generated or analysed during the current study.

## Abbreviations

BSA: Body surface area
eGFR: Estimated glomerular filtration rate
IQR: Interquartile range
LBW: Lean body weight
mCRPC: Metastatic castration-resistant prostate cancer
MIP: Maximum intensity projection
MTV: Metabolic tumour volume
NEMA: National Electrical Manufacturers Association
PET/CT: Positron emission tomography/computed tomography
PSA: Prostate-specific antigen
PSMA: Prostate-specific membrane antigen
RPT: Radiopharmaceutical therapy
SD: Standard deviation
SPECT: Single photon emission computed tomography
SUV: Standardized uptake value
TLA: Total lesion activity
TLF: Total lesion fraction
VOI: Volume of interest
