# Supplementary material for: The tumour sink effect on 68Ga-PSMA-PET/CT in metastatic castration-resistant prostate cancer and its implications for PSMA-RPT: a sub-analysis of the 3TMPO study
Source: Cancer Imaging. 2025 Jul 15;25:91. doi: 10.1186/s40644-025-00910-z (PMC12261768; doi:10.1186/s40644-025-00910-z)
Supplement: Supplementary file 1 — Supplementary Material 1 [file 40644_2025_910_MOESM1_ESM.docx]

**Supplementary materials**

**
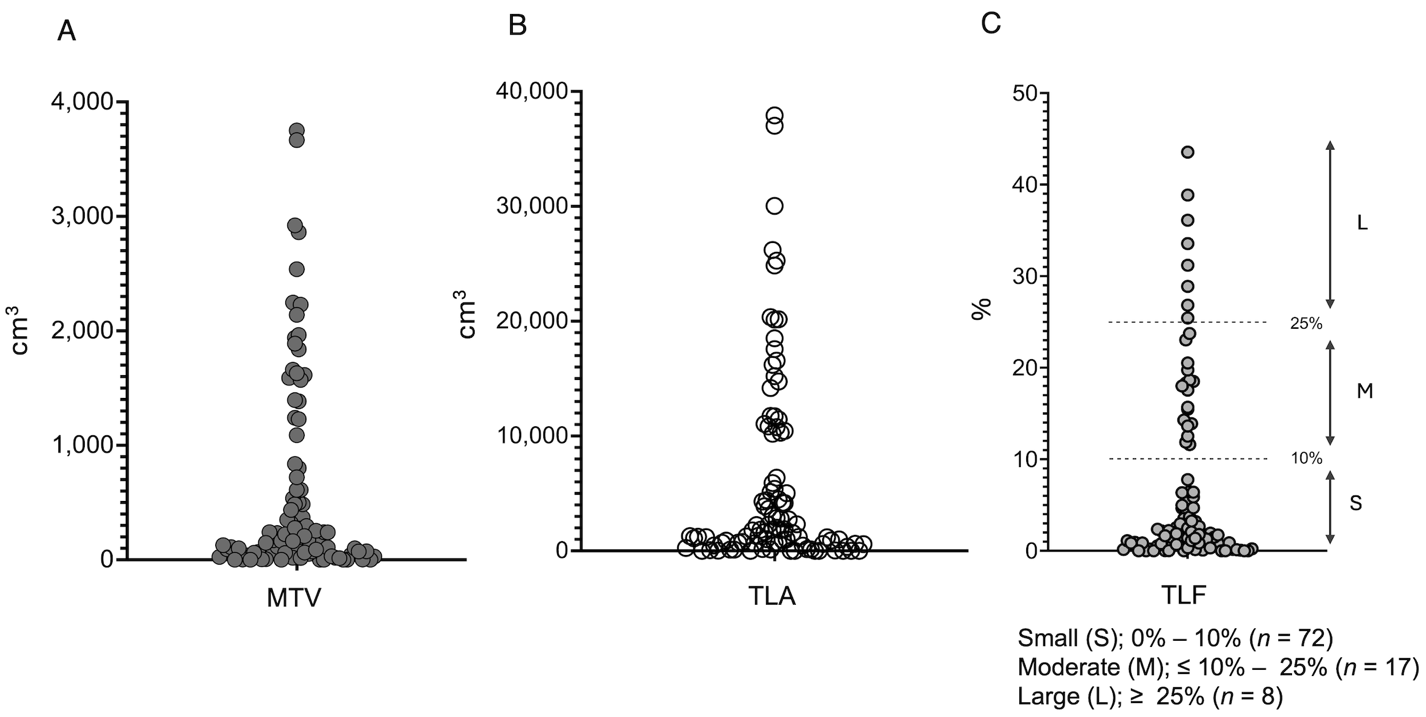
**

**Supplementary Fig. 1** Distribution of molecular tumor volume (MTV; **A**), total lesion activity (TLA; **B**) and total lesion fraction (TLF; **C**) among participants (*n* = 97).

**
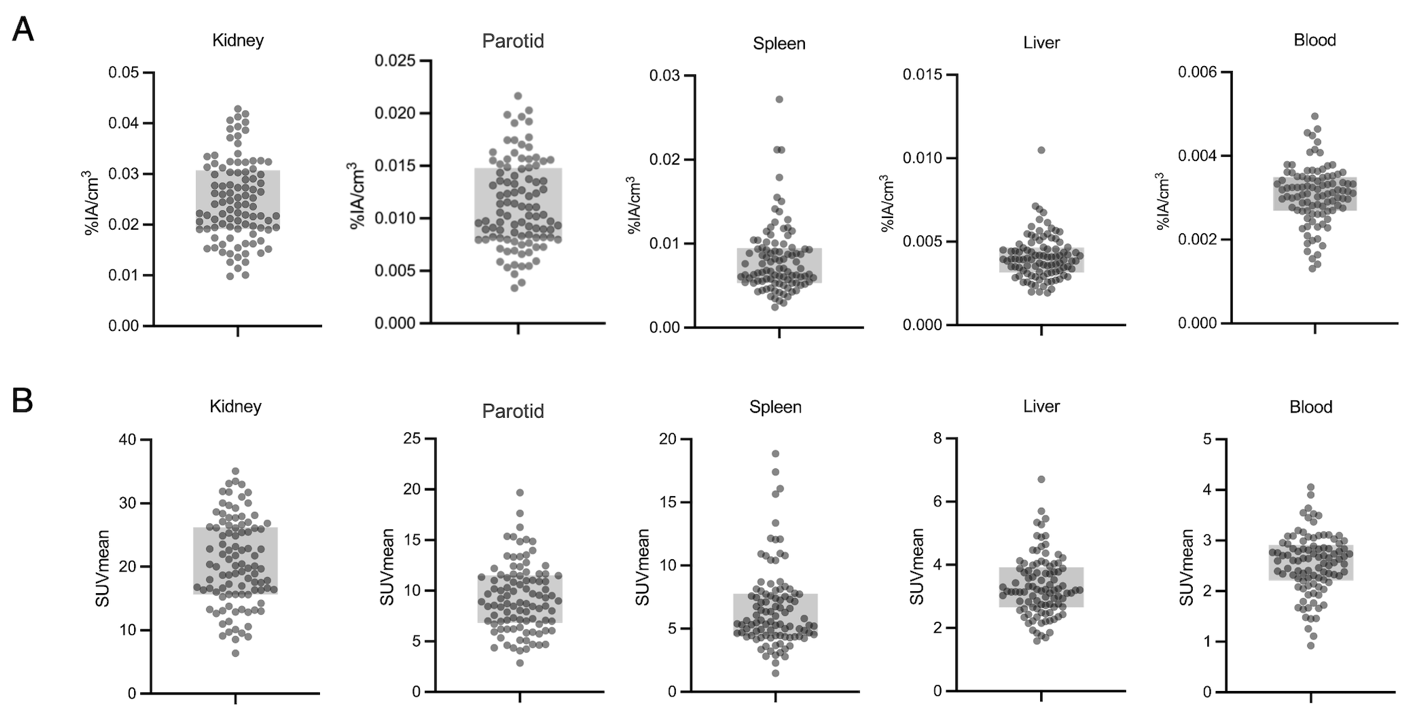
**

**Supplementary Fig. 2** Distribution of healthy tissue uptake, expressed as mean percentage of injected activity per cubic centimeter (%IA/cm^3^; **A**), and mean standardized uptake value (SUV_mean_; **B**) (*n* = 97). Gray boxes represent the interquartile ranges


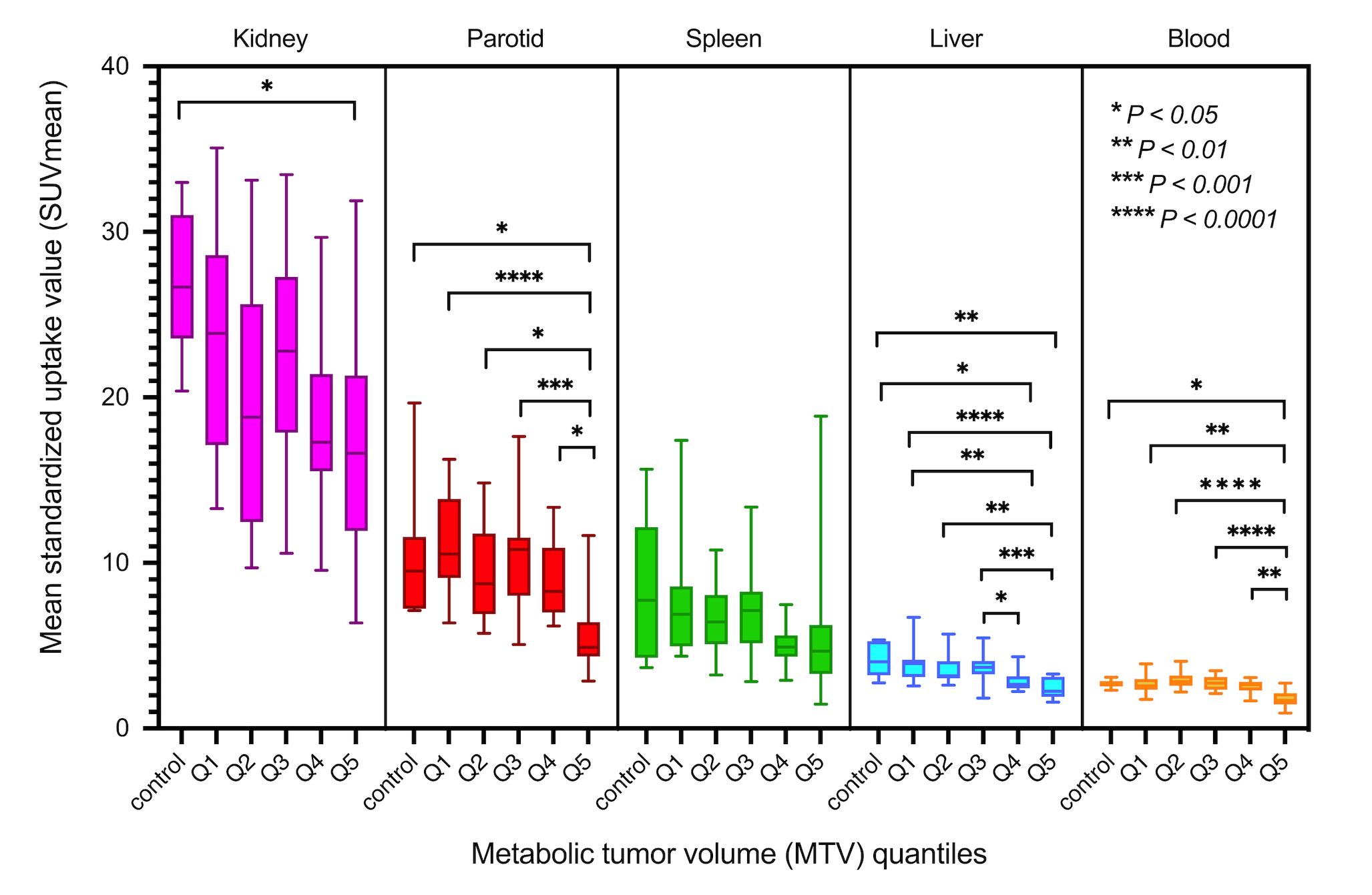


**Supplementary Fig. 3** Healthy tissue uptake (SUV_mean_) for control participants (*n* = 7) and per quintile of MTV (*n* = 90). Control (MTV = 0), Quintile 1 (1.5–63.0 cm^3^), Quintile 2 (66.2–150.6 cm^3^), Quintile 3 (162.8–295.7 cm^3^), Quintile 4 (320.1–1,395.2 cm^3^), Quintile 5 (1572.2–3,750.5 cm^3^). Median, interquartile range (box) and range (whiskers) are shown

**Supplementary Table 1** Spearman’s correlations between healthy tissue uptake (SUV_mean_) and participant characteristics (TLF, body habitus, eGFR, and combinations thereof)

|  | Kidney | | Parotid | | Spleen | | Liver | | Blood | |
| --- | --- | --- | --- | --- | --- | --- | --- | --- | --- | --- |
|  | *r* | *P* | *r* | *P* | *r* | *P* | *r* | *P* | *r* | *P* |
| TLF | **–0.34** | <0.001 | **–0.45** | <0.001 | **–0.35** | <0.001 | **–0.54** | <0.001 | **–0.43** | <0.001 |
| Weight | **0.32** | 0.002 | 0.09 | 0.38 | 0.16 | 0.13 | 0.13 | 0.20 | **0.39** | <0.001 |
| BSA | **0.34** | <0.001 | 0.12 | 0.24 | 0.13 | 0.19 | 0.11 | 0.28 | **0.35** | <0.001 |
| LBW | **0.36** | <0.001 | 0.14 | 0.19 | 0.14 | 0.18 | 0.08 | 0.42 | **0.31** | 0.002 |
| eGFR* | 0.14 | 0.18 | –0.14 | 0.16 | –0.05 | 0.64 | –0.14 | 0.18 | **–0.20** | 0.05 |
| Weight × eGFR | **0.31** | 0.002 | 0.00 | 0.98 | 0.09 | 0.34 | 0.03 | 0.80 | 0.18 | 0.09 |
| BSA × eGFR | **0.27** | 0.007 | –0.04 | 0.70 | 0.03 | 0.74 | –0.03 | 0.74 | 0.02 | 0.87 |
| LBW × eGFR | **0.29** | 0.004 | –0.03 | 0.75 | 0.04 | 0.69 | –0.05 | 0.64 | 0.02 | 0.85 |
| Weight / (1 – TLF) | 0.09 | 0.37 | **–0.20** | 0.05 | –0.06 | 0.59 | **–0.20** | 0.05 | 0.04 | 0.70 |
| BSA / (1 – TLF) | –0.03 | 0.77 | **–0.29** | 0.004 | –0.18 | 0.07 | **–0.34** | <0.001 | –0.15 | 0.15 |
| LBW / (1 – TLF) | 0.00 | 0.93 | **–0.27** | 0.007 | –0.16 | 0.11 | **–0.34** | <0.001 | –0.16 | 0.11 |
| Weight × eGFR / (1 – TLF) | 0.15 | 0.15 | **–0.21** | 0.04 | –0.07 | 0.49 | **–0.21** | 0.04 | –0.09 | 0.40 |
| BSA × eGFR / (1 – TLF) | 0.05 | 0.59 | **–0.30** | 0.004 | –0.15 | 0.15 | **–0.33** | 0.001 | **–0.26** | 0.01 |
| LBW × eGFR / (1 – TLF) | 0.07 | 0.51 | **–0.28** | 0.006 | –0.14 | 0.17 | **–0.32** | 0.001 | **–0.26** | 0.009 |
| *CKD-EPI formula  *r* values in bold are statistically significant.  BSA = body surface area; eGFR = estimated glomerular filtration rate; LBW = lean body weight; SUV_mean_ = mean standardized uptake value; TLF = total lesion fraction. | | | | | | | | | | |
